# H1N1 2009 Pandemic Influenza Virus: Resistance of the I223R Neuraminidase Mutant Explained by Kinetic and Structural Analysis

**DOI:** 10.1371/journal.ppat.1002914

**Published:** 2012-09-20

**Authors:** Erhard van der Vries, Patrick J. Collins, Sebastien G. Vachieri, Xiaoli Xiong, Junfeng Liu, Philip A. Walker, Lesley F. Haire, Alan J. Hay, Martin Schutten, Albert D. M. E. Osterhaus, Steve R. Martin, Charles A. B. Boucher, John J. Skehel, Steve J. Gamblin

**Affiliations:** 1 Erasmus Medical Centre, Department of Virology, Rotterdam, The Netherlands; 2 Medical Research Council, National Institute for Medical Research, The Ridgeway, Mill Hill, London, United Kingdom; 3 MOA Key Laboratory of Plant Pathology, China Agricultural University, Beijing, People's Republic of China; Institut Pasteur, France

## Abstract

Two classes of antiviral drugs, neuraminidase inhibitors and adamantanes, are approved for prophylaxis and therapy against influenza virus infections. A major concern is that antiviral resistant viruses emerge and spread in the human population. The 2009 pandemic H1N1 virus is already resistant to adamantanes. Recently, a novel neuraminidase inhibitor resistance mutation I223R was identified in the neuraminidase of this subtype. To understand the resistance mechanism of this mutation, the enzymatic properties of the I223R mutant, together with the most frequently observed resistance mutation, H275Y, and the double mutant I223R/H275Y were compared. Relative to wild type, K_M_ values for MUNANA increased only 2-fold for the single I223R mutant and up to 8-fold for the double mutant. Oseltamivir inhibition constants (K_I_) increased 48-fold in the single I223R mutant and 7500-fold in the double mutant. In both cases the change was largely accounted for by an increased dissociation rate constant for oseltamivir, but the inhibition constants for zanamivir were less increased. We have used X-ray crystallography to better understand the effect of mutation I223R on drug binding. We find that there is shrinkage of a hydrophobic pocket in the active site as a result of the I223R change. Furthermore, R223 interacts with S247 which changes the rotamer it adopts and, consequently, binding of the pentoxyl substituent of oseltamivir is not as favorable as in the wild type. However, the polar glycerol substituent present in zanamivir, which mimics the natural substrate, is accommodated in the I223R mutant structure in a similar way to wild type, thus explaining the kinetic data. Our structural data also show that, in contrast to a recently reported structure, the active site of 2009 pandemic neuraminidase can adopt an open conformation.

## Author Summary

Recently, a pandemic A/H1N1 influenza virus was isolated from an immune compromised patient with a novel antiviral resistance pattern to the neuraminidase inhibitor class of drugs. This virus had an amino acid change in the viral neuraminidase enzyme; an isoleucine at position 223 was substituted by an arginine (I223R). Patients infected with such a virus leave physicians with reduced antiviral treatment options, since pandemic viruses are naturally resistant to the other class of antivirals, the adamantanes. Previously, we have shown that this mutant virus retains its potential to cause disease and may still be able to spread in the human population.

Here we used enzyme kinetic measurements and crystal structures of the I223R mutant neuraminidase to determine the resistance mechanism of this amino acid change. We found that the I223R change results in shrinkage of the active site of the enzyme. As a result, binding of the neuraminidase inhibitors is affected. In addition, we found that the active site of our pandemic neuraminidase structure, crystallized in the absence of inhibitor, has an extra cavity (150-cavity) adjacent to the active site. Our study could aid in the development of novel inhibitors designed to target the 150-cavity and active site of the enzyme.

## Introduction

Strategies to combat the burden of disease caused by influenza mainly rely on vaccination [1]. However, in cases when vaccine efficacy is low or vaccine is unavailable, for instance during the first months of a pandemic, antiviral drugs are an important line of defense. For individual patients, especially when the immune system is compromised, antiviral therapy may be life saving [2]. Traditionally, both the adamantane and neuraminidase inhibitor class of drugs have been available for prophylaxis and therapy. However, the majority of recently circulating influenza viruses are resistant to the adamantanes, including the A/H1N1 2009 pandemic influenza virus [3]. This leaves neuraminidase inhibitors (NAIs) as the only option.

Stimulated by the first neuraminidase (NA) crystal structures, the NAIs were designed to interact with the active site of all NA types [4], [5]. Because the NA active site is highly conserved, resistant mutants with amino acid changes in the proximity of this active site, were considered likely to be enzymatically compromised and thus predicted to be of marginal epidemiological and clinical significance [6]. Indeed, the first viruses identified as harboring NAI resistant mutations were compromised in their replicative capacity and transmissibility [7]–[9]. Nevertheless, a novel oseltamivir-resistant influenza A/H1N1 variant emerged in the 2007–2008 influenza season and became the dominant virus in some parts of the world [10], [11]. Resistance to oseltamivir was caused by a previously identified, and frequently observed, histidine to tyrosine change in the NA at position 275 (H275Y). In contrast to earlier observations on H275Y mutants, this change did not compromise viral replication or transmissibility in the background of the 2007–2008 H1N1 virus [12]–[14]. Additional mutations in the NA were identified that explained why this resistant virus was able to emerge and become widespread [15], [16].

With the 2009 pandemic, a novel influenza A/H1N1 virus was introduced into the human population [17]. Given that the appearance of NAI drug resistant mutations varies with the type and structure of the neuraminidase that the virus carries, we were interested to identify and characterize novel patterns of resistance in this virus. We identified a novel isoleucine to arginine change at position 223 (I223R) in the NA of a virus isolated from an immune suppressed child on prolonged oseltamivir and zanamivir therapy [18]. In contrast to the frequently observed H275Y change, which causes selective resistance to oseltamivir, the I223R mutation conferred a resistance phenotype against both oseltamivir and zanamivir. Soon after the identification of this first clinical case, the I223R change was found as a single change or in combination with H275Y, in a number of other cases [19], [20]. The I223R/H275Y double mutant proved to have high levels of resistance to oseltamivir. Both *in vitro* studies and in animal models, viruses with I223R and the combination of I223R with H275Y were found not to be compromised in their replication capacity or transmissibility [21], [22].

The structural basis of resistance conferred by the H275Y has been described previously [23]. Here we address the role of the I223R in NAI resistance alone and in combination with H275Y. Both I223R and H275Y changes are near the active site of A/H1N1 pandemic NA. Previously, it was shown that different NA sub types (N1–N9) can be grouped into two genetically distinct groups [24]. The active sites of group-1 NAs (N1, N4, N5, N8) have an extra cavity when crystallized in the absence of inhibitor, because of the ‘open’ conformation of an active site loop, the 150-loop. This loop, containing residues 148–151, closes a cavity adjacent to the active site upon drug binding (150-cavity). In contrast, in group-2 (N2, N3, N6, N7, N9) NA, this loop adopts the closed conformation in the presence or absence of active site ligands. Recently however, a crystal structure for a ligand-free form of the H1N1 pandemic NA was reported, which showed the active site in a closed conformation [25]. More recently, NMR studies on the pandemic neuraminidase have considered this difference and suggested that the neuraminidase prefers to adopt an open conformation [26]. Our results support this suggestion by indicating that a ligand-free form of the pandemic neuraminidase adopts an open conformation. Together with this structure we present the structures of the I223R mutant neuraminidase in complex with oseltamivir and zanamivir to investigate the resistance mechanism of the I223R mutant neuraminidase. We interpret our results of enzyme kinetic measurements in relation to the structures of the complexes.

## Results/Discussion

The NA studied here originated from a pandemic influenza A/H1N1 virus which was isolated from an immune suppressed child on oseltamivir and intravenously (IV) administered zanamivir antiviral therapy. It contained the mutation I223R [18]. Since this change has been reported both as a single mutation as well as in combination with the substitution H275Y [19], virus recombinants were constructed containing the wild type NA, the I223R or H275Y single mutants and the I223R, H275Y double mutant.

### Enzyme kinetics of the I223R and H275Y mutant neuraminidases

To study the effects of the single mutations I223R and H275Y, and the double mutant on the enzymatic properties of NA, Michaelis-Menten (K_M_) constants were determined using MUNANA as a substrate. Relative to wild type NA, K_M_ values increased marginally for the I223R and H275Y single mutants. There was a maximum 8-fold increase for the double mutant (Table 1). In the 2007–2008 influenza H1N1 season, naturally occurring H275Y variants had emerged, which were not attenuated in virus growth or transmissibility. Our observations suggest that, as was observed with the non-attenuated H275Y variants in the 2007–2008 season, the occurrence of viable NAI resistant pandemic influenza A/H1N1 viruses is not an unlikely event.

**Table 1:** Kinetic parameters and oseltamivir/zanamivir drug binding for 2009 pandemic influenza A/H1N1 neuraminidase mutants.

			Oseltamivir			Zanamivir		
Virus	PDB ID	K_M_ *	K_I_ (nM)	k_on_ (µM^−1^ s^−1^)	10^3^×k_off_ (s^−1^)	K_i_ (nM)	k_on_ (µM^−1^ s^−1^)	10^3^×k_off_ (s^−1^)
Wild type	4B7Q/4B7R	1.0	0.23 (0.12)	3.10 (0.12)	0.75 (0.20)	0.18 (0.03)	1.31 (0.15)	0.32 (0.10)
I223R	4B7M/4B7J/4B7N	2.1	11.1 (1.10)	1.04 (0.09)	11.1 (0.90)	1.65 (0.40)	0.36 (0.12)	0.78 (0.12)
H275Y	ND**	2.6	145 (32)	ND	ND	0.53 (0.06)	1.02 (0.04)	0.76 (0.21)
I223R/H275Y	ND	8.1	1750 (150)	ND	ND	3.92 (0.15)	0.15 (0.01)	0.72 (0.24)

Measurements were performed in triplicate and values in parentheses are standard deviations from the mean.

* K_M_ values presented relative to wild type.

K_M_ value for wild type = 28.0 µM.

** ND = Not determined.

The sensitivity to oseltamivir and zanamivir was determined for the single and double mutants. By comparison to the oseltamivir inhibitor constant (K_I_) of wild type NA, the K_I_ of the I223R mutant increased 48 times and the I223R/H275Y double mutant K_I_ more than 7500 times. The change in the K_I_ for I223R (48-fold) was largely accounted for by an increased dissociation rate constant for the enzyme-inhibitor complex (15-fold) rather than by a reduced association rate constant (k_on_) (3-fold). In the case of zanamivir, the K_I_ increased 9-fold for the single and 22-fold for the double mutant relative to wild type. The change in the K_I_ for I223R (9-fold) was in this case accounted for by a reduced association rate constant (4-fold) and a slightly increased dissociation rate constant (2-fold). Thus the I223R mutation has a markedly greater effect on NA inhibition by oseltamivir than by zanamivir.

Although the K_M_-values for MUNANA are increased about equally for both the I223R and H275Y single mutants, the change in oseltamivir inhibitor constant for the H275Y mutant relative to wild type, is approximately 10 times greater than for the I223R mutant (Table 1). This may explain why the H275Y change may be the more likely resistance change in oseltamivir monotherapy and of course, to date, the H275Y mutation is the most frequently detected oseltamivir resistance mutation.

### The active site of the H1N1 pandemic neuraminidase has an open conformation

**Figure 1:**
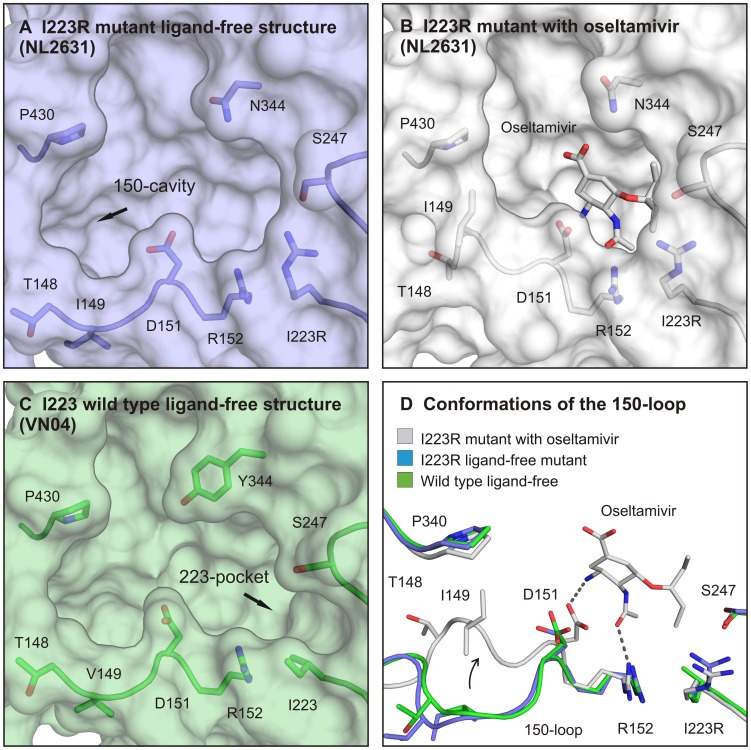
Crystal structures of H1N1 2009 pandemic neuraminidase reveal the open conformational state of its active site. Panel A presents the active site of a 2009 pandemic neuraminidase (PDB ID code 4B7M) in an open conformation. This is the preferred state in the absence of neuraminidase inhibitor. When a protein/inhibitor complex is formed, a flexible loop (150-loop) closes the 150-cavity (panel B). Both the open and closed neuraminidase structures lack a smaller cavity near position 223, due to an isoleucine to arginine change (I223R). This pocket is still present in the wild type N1 neuraminidase (PDB ID code 2HTY [24]) structure of H5N1 (panel C). Further narrowing of the active site by the I223R change causes antiviral resistance to both oseltamivir and zanamivir. In panel D, an overlay of the 150-loop is presented showing important amino acids in close proximity to the active site. An aspartic acid D151 in the 150-loop and arginine R152 in the 150-loop make hydrogen bond contacts with oseltamivir (or zanamivir) and stabilize the closed conformation of the neuraminidase.

Crystals of wild type and mutant neuraminidases were grown in the absence or presence of the oseltamivir and zanamivir neuraminidase inhibitors. The crystals yielded high-resolution diffraction data and the structures were solved by molecular replacement and refined by standard procedures (Table 2.) The first striking feature from these data is that the 150-loop in the ligand-free I223R NA adopts an open conformation, as seen in the first reported N1 structure [24], but in contrast to the closed conformation of this loop seen in the recently reported crystal structure of the 2009 pandemic A/H1N1 NA (Figure 1A) [25]. Recent NMR experiments likely clarify the apparent discrepancy in these different studies by showing that the 150-loop is flexible and that an equilibrium between the open and closed conformations exists in solution [26]. Inspection of our current structure shows that a phosphate ion, from the crystallization buffer, interacts with lysine 150 to stabilize the open conformation. We speculate that the different crystallization conditions used in the previous pandemic N1 study led to the stabilization of the closed form [25]. The exact function of the open 150-loop in group-1 neuraminidases is unknown, but opening and closing of the loop may have evolved for natural sialo-glycan substrates to fit into the active site. The open conformation of the 150-loop (residues 148–151) generates an additional cavity at the edge of the active site that we have previously called the 150-cavity (Figure 1A), which we have suggested to be an additional target site for neuraminidase inhibitor design [24], [27].

**Table 2:** Data collection and refinement statistics.

**Virus**	NL2631	NL2631	NL2631	Cal07	Cal07
**NAI**	-	Oseltamivir	Zanamivir	Oseltamivir	Zanamivir
**PDB ID code**	4B7M	4B7J	4B7N	4B7R	4B7Q
**Amino acid differences** *	V106I, I223R, N248D	V106I, I223R, N248D	V106I, I223R, N248D	-	-
**Data Collection**					
Wavelength (Å)	0.9173	0.9763	0.97625	0.97950	0.97630
Space group	C222_1_	P42_1_2	P42_1_2	P2_1_2_1_2_1_	P2_1_2_1_2_1_
Cell dimensions					
*a, b, c* (Å)	118.6, 162.5, 118.9	118.6, 118.6, 67.8	118.4, 118.4, 68.4	83.4, 149.0, 166.8	82.7, 148.8, 166.6
*α, β, γ* (°)	90, 90, 90	90, 90, 90	90, 90, 90	90, 90, 90	90, 90, 90
Resolution (Å)	30.0-2.5 (2.61-2.50)	83.8-2.4 (2.55-2.42)	83.7-2.84 (3.00-2.84)	30.0-1.90 (1.99-1.90)	82.65-2.73 (2.80-2.73)
*R_merge_*	14.2 (60.8)	13.0 (41.6)	15.7 (48.1)	12.5 (72.8)	20.5 (53.8)
*I/σI*	9.3 (2.1)	11.4 (4.5)	15.3 (6.0)	13.5 (3.0)	6.1 (2.5)
Completeness (%)	93.3 (92.0)	100 (100)	100 (100)	99.7 (98.0)	99.2 (99.0)
Multiplicity	5.6 (4.6)	10.8 (10.5)	13.3 (13.3)	6.4 (4.8)	6.8 (6.4)
Unique reflections	37388	19102	11923	163254	55091
**Refinement**					
Resolution (Å)	95.8-2.5	84-2.42	83.6-2.84	111-1.9	82.6-2.7
No. reflections	27395	17197	11333	154943	52204
*R_work_/R_free_*	20.5/23.7	22.8/27.6	18.5/22.4	18.4/20.4	22.4/27.1
R_free_ test set size (%)	4.9	5.1	4.8	5.0	5.1
R_free_ test set count	1412	931	567	8190	2790
No. atoms	6367	3196	3179	13079	12721
B-factor	16.5	13.1	12.7	28.6	35
R.m.s deviations					
Bond lengths (Å)	0.007	0.005	0.006	0.007	0.0054
Bond angles (°)	1.437	0.995	1.037	1.281	0.9922
**MolProbity**					
MolProbity, clash score	0.16 (100^th^ percentile)	0.33 (100^th^ percentile)	0.65 (100^th^ percentile)	0.08 (100^th^ percentile)	0.33 (100^th^ percentile)
MolProbity score	1.00 (100^th^ percentile)	1.01 (100^th^ percentile)	1.09 (100^th^ percentile)	0.76 (100^th^ percentile)	1.07 (100^th^ percentile)
Ramachandran favored (%)	95.6	95.32	96.35	96.49	95.26
Residues with bad bonds (%)	0.0	0.78	0.0	0.0	0.0
Residues with bad angles (%)	0.0	0.26	0.0	0.0	0.06

* Amino acid differences as compared to Cal07.

### Changes in oseltamivir binding by the I223R change

**Figure 2:**
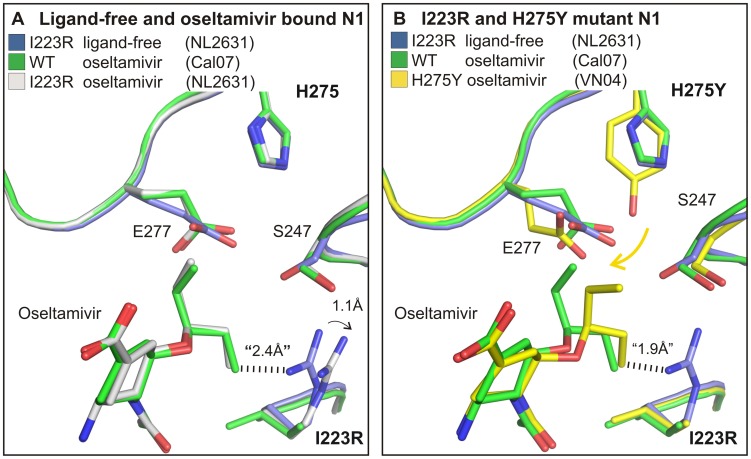
Antiviral resistance mechanism of the I223R mutant. In panel A, an overlay of three pandemic neuraminidase structures is presented: I223R mutant ligand-free structure (blue, NL2631, PDB ID code 4B7M) and both wild type (green, Cal07, PDB ID code 4B7R) and I223R mutant (white, Cal07, PDB ID code 4B7J) oseltamivir complexes. The key amino acid residues involved in I223R antiviral resistance pattern are displayed. Binding of oseltamivir to the active site is inhibited by arginine 223 (R223) and serine 247 (S247). The side chain of S247 points towards the active site in the I223 mutant. Oseltamivir binding to the I223R mutant involves reorientation of both R223 and S247 residues. In panel B, an overlay is presented of an H275Y mutant neuraminidase structure in complex with oseltamivir (yellow, VN04, PDB ID code 2CL0 [23]) with two I223R mutant neuraminidase crystal structures: The ligand-free structure (blue, NL2631, PDB ID code 4B7M) and the structure in complex with oseltamivir (green, NL2631, PDB ID code 4B7J). The resistance mechanisms of the H275Y and I223R mutants act synergistically to gain enhanced levels of oseltamivir resistance.

In the wild type N1 neuraminidase structure, a small pocket, the “223-pocket” (Figure 1C), also adjacent to the active site, but distinct from the 150-cavity, contains two water molecules. In the I223R neuraminidase, this pocket is occupied by the side chain of R223. In displacing the two water molecules seen in the wild-type structure, the arginine makes a hydrogen bond with the side chain of serine 247 (S247). As a result, the side chain of S247 adopts a different rotamer and is oriented towards the active site which enables it to make a second hydrogen bond with glutamic acid 277 (E277). These changes in the structure of the I223R neuraminidase result in shrinkage at the edge of the active site pocket that accommodates the hydrophobic pentoxyl group of oseltamivir. Thus, oseltamivir binding to the active site of I223R neuraminidase requires the reorientation of the S247 and R223 side chains. Moreover, the reorientation of the side-chain of E277, which is required for oseltamivir binding to wild-type neuraminidase, is presumably made even less energetically favorable in the I223R mutant by the need to disrupt the hydrogen bond between S247 and E277. Thus the binding of oseltamivir to the active site of the I223R mutant results not only in changes in the conformation of the side chain of S247 but also in the side-chain of R223 being translated about 1.1 Å out of the active site compared to the mutant ligand-free structure (Figure 2A).

S247 therefore plays an important role in the decreased sensitivity of the I223R neuraminidase to inhibitors. Interestingly, a serine to asparagine change at position 247 (S247N) has also been linked to oseltamivir resistance [28]. Like the I223R change, the S247N mutation also causes enhanced resistance to oseltamivir when accompanied by the H275Y change. Both the single and the S247N/H275Y double mutant viruses were found not to be compromised in a ferret model suggesting that the mutant neuraminidases retain most of their normal activity [29]. An asparagine at position 247, an amino acid with a larger polar side chain, can also affect the size of the hydrophobic pocket. Therefore, although there is no structural information in that case we speculate that, like the I223R change, the resistance mechanism of the S247N change also may involve shrinkage of the hydrophobic pocket.

### Structural changes in the I223R/H275Y double mutant

The mechanism whereby the H275Y mutant neuraminidase binds substantially more weakly to oseltamivir than wild type has been described previously [23]. In brief, the introduction of the bulkier tyrosine residue perturbs the position and the reorientation of the acidic side chain of E277 required for oseltamivir binding, such that the hydrophobic pentoxyl substituent of oseltamivir is translated out of the active site towards the pocket occupied in the I223R structure by the side chain of the arginine residue (Figure 2B). The I223R mutation, therefore, appears to restrict the alternative position that the pentoxyl substituent adopts in the H275Y mutant. Furthermore, the binding of oseltamivir by the I223R/H275Y double mutant becomes even less energetically favorable than by either of the two single mutant proteins. Our structural data therefore provide an explanation for the 1750-fold poorer binding of oseltamivir by the I223R/H275Y double mutant by comparison with the wild-type neuraminidase (Table 1).

### The polarity of the zanamivir glycerol substituent correlates with the smaller effect of the I223R mutation on zanamivir binding

**Figure 3:**
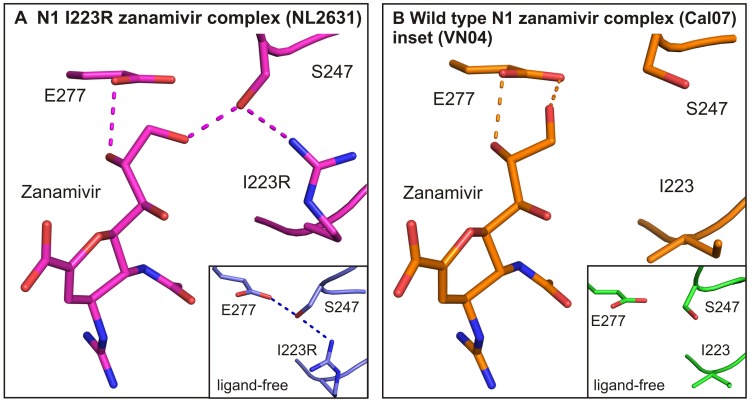
The polarity of zanamivir enables inhibition of the I223R mutant. In panel A, hydrogen bond contacts are presented (dotted lines), which are formed between zanamivir and active site residues in the I223R mutant (purple, PDB ID code 4B7N). The insert displays the interactions between the same residues in the I223R mutant ligand-free structure (blue, PDB ID code 4B7M). In panel B, active site residues and hydrogen bonds are displayed, which are formed in the wild type zanamivir structure complex (brown, PDB ID code 4B7Q). The insert displays the I223, S247 and E277 residues in the wild type ligand-free structure.

Our structural studies help to explain how the single I223R and I223R/H275Y double mutant neuraminidases bind zanamivir almost as well as wild type. Instead of the hydrophobic pentoxyl substituent present in oseltamivir, zanamivir possesses the same polar glycerol substituent as sialic acid (Figures 2 and 3). Consequently, binding of zanamivir to the active site of neuraminidase does not require the reorientation of the side chain of E277. In the case of the wild type neuraminidase, the glycerol moiety of zanamivir forms two hydrogen bonds with E277. This interaction is unaffected in the H275Y mutant (Figure 3). In the I223R single or double mutant neuraminidases, somewhat different interactions are made by the glycerol moiety: one hydrogen bond is formed with E277 and another hydrogen bond is formed with S247 (Figure 3A). To facilitate zanamivir binding in this way R223 is reoriented only marginally and it still retains the R223 hydrogen bond with S247 (Figure 3).

The plasticity of the pandemic neuraminidase active site, especially around the hydrophobic pocket, allows the emergence of drug resistant influenza variants. In addition to the I223R change, isoleucine 223 changes to valine (I223V) and lysine (I223K) accompanying the H275Y change have also been reported [20]. This further indicates that this residue plays an important role in neuraminidase inhibitor resistance and stresses the importance of a detailed understanding of the mechanism of resistance. Since the single I223R change described here resulted in only a 9-fold increase in the K_I_ for zanamivir, it is interesting to consider why the I223R mutant virus persisted in the IV zanamivir treated immunocompromised patient. As previously suggested [30], suboptimal drug levels in the patient's respiratory tract, due to the route of administration (IV versus inhaled) as well as the immune status of the patient may have contributed to the emergence and persistence of the I223R mutant virus.

## Materials and Methods

### Antiviral compounds and recombinant viruses

Hoffmann-La Roche Ltd. (Switzerland) kindly provided oseltamivir carboxylate. Zanamivir was kindly provided by GlaxoSmithKline (the Netherlands). Recombinant influenza viruses were generated by reverse genetics essentially as previously described [31]. Each virus contained seven segments of A/WSN/33 and the neuraminidase gene of either A/Netherlands/2631_1202/2010 (NL2631), A/California/07/09 (Cal07) or A/Vietnam/1203/04 (VN04). Recombinant viruses were propagated in embryonated chicken eggs. Allantoic fluids were harvested after 48 hrs and cleared from debris by centrifugation at 3000 rpm for 15 minutes. They were then used directly to measure enzyme kinetics or further processed to purify the neuraminidase for crystallography.

### Neuraminidase activity measurements

Michaelis-Menten constants (K_M_) were determined at 37°C as previously described [23], using standard initial rate measurements with virus diluted in 32.5 mM MES buffer (pH 6.4), 5 mM CaCl_2_ and fluorescent substrate 2′-(4-methylumbelliferyl1)-α-D-N-acetylneuraminic acid (MUNANA) concentrations ranging from 2.0 to 200 µM. Inhibition constants (K_I_) were determined by following the reduction in MUNANA hydrolysis rate following addition of inhibitor to a virus/MUNANA mixture approximately 100 s after initiation of the reaction. K_I_ was then calculated using equation (1) [32]: 
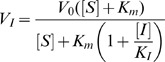
(1)where V_I_ is the new steady state hydrolysis rate in the presence of inhibitor at concentration [I], V_0_ is the rate in the absence of inhibitor, [S] is the MUNANA concentration, K_M_ is the Michaelis-Menten constant and K_I_ is the dissociation constant for inhibitor binding. The kinetic parameters for inhibitor binding were determined by analyzing the exponential approach to V_I_ using the following equation [33]:
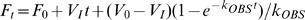
(2)where F_0_ and F_t_ are the fluorescence signals at time zero and time t, and k_OBS_ is the first-order rate constant. Association (k_on_) and dissociation (k_off_) rate constants for inhibitor binding were then determined by plotting k_OBS_ versus [I] using the following equation [34]:
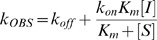
(3)In most cases the kinetically determined K_I_ values (k_off_/k_on_) agree well with those determined using equation (2). Measurements were performed in triplicate using three different inhibitor concentrations.

### Neuraminidase purification

Recombinant virus was harvested from clarified allantoic fluid by centrifugation at 6000 rpm overnight at 4°C and resuspended in 10 mM TRIS buffer, (pH 8.0), 150 mM NaCl (TRIS-buffer). Virus was then layered over a continuous sucrose gradient (15–40% sucrose in TRIS-buffer) and centrifuged at 25.000 rpm for 45 minutes at 4°C. The virus-containing fraction was collected and virus was pelleted by dilution of the remaining sucrose with Tris-buffer and centrifugation at 27.000 rpm for 90 minutes at 4°C. The purified virus was resuspended in Tris-buffer. Next, glycoproteins were released from the virus by bromelain (Sigma-Aldrich) digestion for 1 hour at 37°C and digested viruses were pelleted by centrifugation at 55.000 rpm for 10 minutes. A protease inhibitor tablet was added to the NA containing supernatant to prevent further protein degradation (Roche). NA was purified by layering the supernatant over a sucrose gradient (5–25% sucrose in 10 mM TRIS buffer (pH 8.0) and centrifugation at 38000 rpm for 18 hours at 4°C. Subsequently, NA-containing fractions were pooled and further purified using an Q-15 anion-exchange column (Sartorius). Finally, buffer was changed by overnight dialysis against Tris-buffer supplemented with 5 mM CaCl_2_ and neuraminidase protein was concentrated 6 mg/ml using a 50 kD vivaspin column (Sartorius) for crystallization experiments.

### Protein crystallography

Neuraminidase crystals were obtained by vapor diffusion from sitting drops dispensed with an Oryx 8 robot (Douglas Instruments). The drops consisted of 100 nl of protein in the absence or presence of 1 mM inhibitor (oseltamivir or zanamivir) mixed with 100 nl of reservoir solution. The reservoir solution of the I223R ligand-free crystal consisted of 20% PEG1000, 0.6 M Ammonium Phosphate and 0.1 M Sodium Acetate (pH 4.6). The reservoir solution of the I223R crystal in complex with zanamivir consisted of 18% PEG3350, 0.2 M Sodium Fluoride and 0.1 M bis-TRIS Propane buffer (pH 6.5). The reservoir solutions of the other crystals consisted of 15% PEG3350, 0.1 M bis-TRIS propane and 0.1 M sodium acetate buffer (pH 4.6). Crystals were transferred into a cryoprotectant that consisted of reservoir solution supplemented with 20% (v/v) ethylene glycol before flash freezing in liquid nitrogen. Data sets were recorded on an ADSC Q315 CCD, Pilatus 6M-F and SLS/Dectris Pilatus miniCBF detectors at the Diamond light source (Oxford, UK). Diffraction images were integrated using iMOSLFM [35] or DENZO and scaled with SCALA [36] or SCALEPACK for the ligand-free I223R structure. Neuraminidase structures were solved by molecular replacement with PHASER [37] using the wild type structure (protein databank (PDB) identification (ID) code 2HU4) as the initial search model. Refinement was performed using Refmac5 [38] or PHENIX Refine [39]. Manual model building was done using Coot [40], structure validation was assessed with MOLPROBIDITY [41] and figures were created using Pymol (http://pymol.sourceforge.net/).
